# The Analysis of Efficacy and Failure Factors of Uterine Artery Methotrexate Infusion and Embolization in Treatment of Cesarean Scar Pregnancy

**DOI:** 10.1155/2013/213603

**Published:** 2013-10-24

**Authors:** Xiao An, Xu Ming, Ke Li, Jingbing Wang

**Affiliations:** Department of Vascular Surgery and Interventional Radiology, Shanghai First People's Hospital, School of Medicine, Shanghai Jiao Tong University, No. 100 HaiNing Road, HongKou District, Shanghai 200080, China

## Abstract

*Objectives*. This study observes therapeutic efficacy of uterine artery embolization combined with MTX infusion which terminates cesarean scar pregnancy (CSP) and induces three factors which probably relate to failure. *Methods*. Twenty-three CSP patients were treated with combined uterine artery MTX infusion and embolization. Among them six patients with severe hemorrhage were immediately treated with interventional operation. Clinical effects were estimated by symptoms, serum *β*-hCG, ultrasound, and MR. *Results*. Interventional treatments were technologically successful in 22 patients except one. Immediate hemostasis was achieved in all 6 patients with massive colporrhagia. No occurrence of infection and uterine necrosis was observed, but 12 women suffered abdominal pains. Nineteen patients' uteri were preserved, whereas four underwent hysterectomy eventually. *Conclusions*. Transcatheter arterial chemoembolization is effective to treat high-risk CSP in preference to hysterectomy. To achieve more successful outcomes, three factors should be highlighted: adequate MTX dosage, appropriate embolic material, and complete embolization of target arteries that supply blood to embryo in the scar.

## 1. Introduction

Caesarean scar pregnancy (CSP), first reported by Larsen and Solomon in 1978 [1], is one of the rarest forms of ectopic pregnancy in which a gestational sac implants in the myometrium at the site of a previous cesarean section. The ratio of CSP to pregnancy was from 1 : 1800 to 1 : 2216 in women with at least one previous cesarean section, and CSP comprised 6.1% of all ectopic pregnancies [2, 3].

However, the incidence of CSP has risen rapidly during the past ten years due to increased rate of cesarean section worldwide [4]. Moreover, with the fast pace of technological advancement, early CSP cases could be easily detected by using ultrasound technique and other imaging technology.

Undoubtedly CSP is an unexpected and dangerous situation with potential risks of uterine rupture, profuse bleeding, and other life-threatening complications when pregnancy advances. Therefore termination of pregnancy in the first trimester is strongly recommended [5]. 

The treatments of CSP include systemic or local MTX administration, dilatation and curettage (D&C), uterine artery embolization (UAE), conservative surgery such as local resection of the ectopic gestational mass or suction curettage by operative hysteroscopy, and finally hysterectomy [6]. 

Among them, UAE combined with local MTX infusion is regarded as an effective nonsurgical therapy to eliminate the gestational sac and meanwhile to preserve fertility [7]. We treated twenty-three CSP patients with this combined interventional management in three years. However, some patients failed to preserve their uteri eventually. In the present work, a single-center retrospective analysis was carried out to observe therapeutic efficacy and identify which factor might cause the failure. 

## 2. Methods

Twenty-three women with postmenstrual age of 6 to 12 weeks were admitted to Shanghai First People's Hospital, School of Medicine, Shanghai Jiao Tong University from 2010 to 2012. The mean age was 31.4 ± 5.8 years. All of them had lower segment caesarean section, and mean interval between the previous caesarean section and the interventional operation was 4.2 years (11 months to 13 years). 

All patients were diagnosed as CSP based on clinical symptoms, serum *β*-human chorionic gonadotropin (*β*-hCG) level, cesarean delivery history, gynecologic examination, transvaginal ultrasound, and/or magnetic resonance imaging (MRI) [8]. 

Eight women had been injected intramuscularly with 1 mg/kg MTX prior to interventional operation in the 2010 and early 2011. The other patients no longer received MTX administration before the DSA operations after clinic feedback.

Informed consents were obtained from all patients before UAE treatment began. This retrospective study was approved by Shanghai First People's Hospital (School of Medicine, Shanghai Jiao Tong University) Service Ethics Committee (number 2012K006). Ethical procedures were in compliance with the Helsinki Declaration.

During interventional operation under digital subtraction angiography (DSA), bilateral uterine arteries were super-selectively catheterized by 4 Fr Yashiro catheter (Terumo, Tokyo, Japan) or Cobra 2 type catheter (Cook, Bloomington, USA), and sometimes a 2.7 Fr microcatheter (Terumo, Tokyo, Japan) with a 0.018 inch guidewire (Terumo, Tokyo, Japan) was introduced into the target arteries by coaxial method. Currently extensive uteroplacental hypervascularity with numerous tortuous branching on angiography indicated the formation of new blood vessels-gestational sac. In some cases there were extravasations of contrast media which are the sure signs of active bleeding.

**Figure 1:**
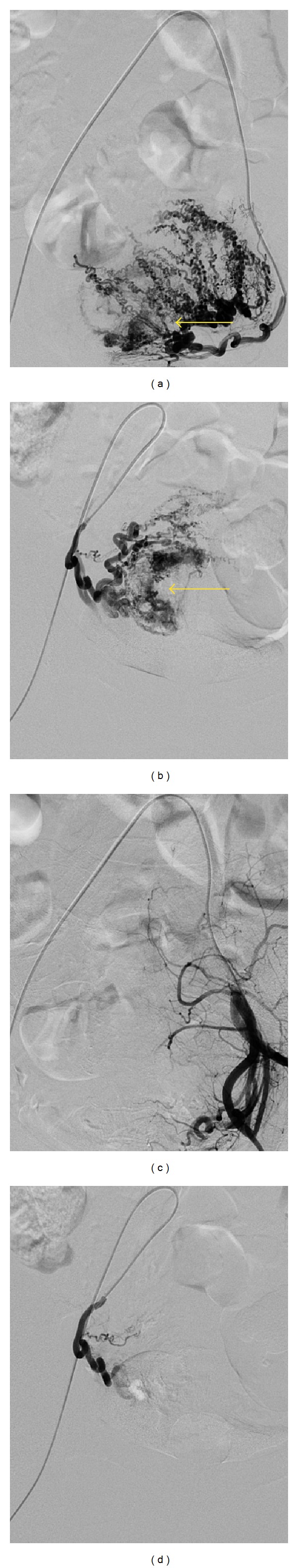
(a, b) Selective bilateral uterine arteries DSA showing persistence of the prominent vessel in the lower anterior uterine wall and gestational sac measuring about 4 cm × 4 cm (arrow). (c, d) Postembolisation angiogram showing complete occlusion of the bilateral uterine artery and gestational sac disappeared.

MTX at dosage of 75 mg/m^2^ was dissolved in 50 mL normal saline and then infused into the uterine arteries in fifteen minutes. Afterwards, the target arteries were embolized with gelatin sponge particles bilaterally. Successful embolization was defined as the total disappearance of the vascularity of the gestational sac on arteriography (Figure 1). 

The patients were followed up by measuring serum *β*-hCG level on day 1, 3, and 5 after-operation and then at weekly intervals until the *β*-hCG level reverted to normal. Transvaginal ultrasonography was used to measure the gestational sac volume and the area vascularization on day 7, 14, and subsequently once a month. Operation-associated complications were also noticed.

## 3. Results

**Figure 2:**
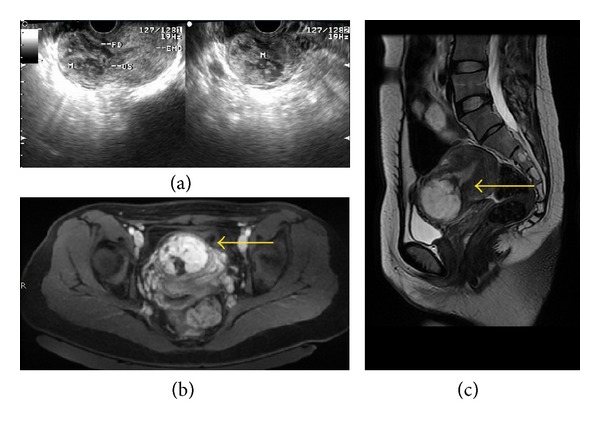
(a) Transvaginal gray-scale ultrasonography revealing an irregular gestational cystic mass (FD) located within the isthmic area of the lower anterior wall of the uterus and protruding toward the uterine cavity. (b) Obvious and homogenous enhancement of the gestational sac after Gd-DTPA administration on axial T1-weighted imaging (arrow) without enhancement of the inner content. (c) Huge gestational sac (4.9 cm × 4.0 cm × 4.4 cm) showing high signal intensity on sagittal T2-weighted imaging (arrow) with low signal intensity inside.

On admission, mean serum *β*-hCG of these 23 CSP patients was 22,530 ± 3,488 mIU/mL (mean ± SD). Ultrasound or MRI revealed that gestational sacs varied in size from 4.9 cm × 4.0 cm × 4.4 cm to 0.9 cm × 0.4 cm × 0.6 cm which implanted at the sites of prior cesarean section scar. For example, a huge gestational sac (4.9 cm × 4.0 cm × 4.4 cm) was illustrated in Figure 2, which was localized in the previous cesarean section scar displaying through transvaginal ultrasound and MRI imaging. 

The most common clinical manifestation of CSP was amenorrhoea (23/23, 100%) and followed by vaginal bleeding (18/23, 78.26%). Six patients who had severe hemorrhage were promptly treated by uterine artery chemoembolization to control the bleeding. Other haemodynamically stable women underwent interventional management within four days after diagnosis. 

UAE operations were technically successful in 22 cases (22/23, 95.7%). Only one patient was managed apart from embolotherapy because her right uterine artery was not obviously enlarged and excessively tortuous which caused difficulty in superselectively catheterizing target vessel. As a result, only MTX solution was injected into the right side, while MTX infusion plus embolization was carried out on the left artery in the procedure. Fortunately after arterial interventional treatment, her serum *β*-hCG level descended gradually with additional injection of intramuscular MTX. This woman's result was satisfactory that ectopic embryo was killed and she retained the fertility. 

Immediate hemostasis was achieved in all six patients with severe hemorrhage by emergency UAE (6/6, 100%). Twelve (12/23, 52.2%) patients suffered minor complications such as pelvic pain during the first two days after the interventional management, but the complications were controlled with proper treatment and resolved within a few days. No infection, myelosuppression, liver and kidney dysfunction, missed embolization, or uterine necrosis was observed.

After UAE, serum *β*-hCG level decreased to 5,224 ± 2,783 mIU/mL in eleven patients within the first 7 days and reached to normal within 42 days. Their sonography showed the reducing sizes of gestational cystic mass at the site of previous cesarean scar during the first two weeks, and they are completely involuted in two months. 

There were three special cases. The first patient required the laparotomy and uterine repair because her gestational sac measured 4.9 cm × 4.0 cm × 4.4 cm on MR (Figure 2). Despite the downward trend in *β*-hCG was observed after the uterine artery MTX infusion and embolization, the patient urgently demanded further surgical treatment to get rid of embryo. The following operation was uneventful, and the result was satisfactory.

**Figure 3:**
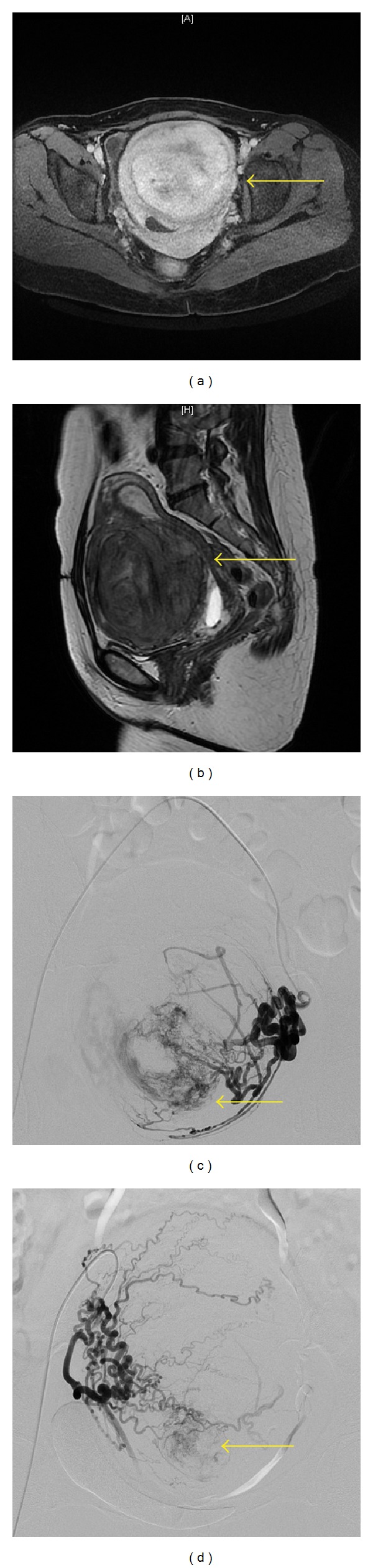
(a) Obvious and homogenous enhancement of the hysteromyoma and gestational sac after Gd-DTPA administration on axial T1-weighted imaging (arrow). (b) Giant hysteromyoma (9.9 cm × 9.5 cm × 9.8 cm) showing almost equal signal intensity on sagittal T2-weighted imaging (arrow). (c, d) Selective bilateral uterine arteries DSA showing persistence of the prominently enlarged and tangled vessels in the uterine and gestational sac measuring about 2.5 cm × 2 cm (arrow).

The second one was diagnosed as CSP complicated with giant hysteromyoma (9.9 cm × 9.5 cm × 9.8 cm) on MR (Figure 3). Forty days after successful compound interventional management, she received myomectomy and laparotomy repair. In the surgery, a 5 cm dead fetus was removed from the scar area. 

Serum *β*-hCG level in the third patient reduced to 6,984 mIU/mL in 4 day, but she was anxious and complained of abdominal discomfort. After careful examination and good communication, she underwent dilatation and curettage on the fifth day after interventional radiology procedure. The D&C operation was uncomplicated, and serum *β*-hCG level declined to normal within 3 days.

The other nine patients received an additional injection of intramuscular MTX (0.5 mg/kg) every two days after DSA operation due to these *β*-hCG levels continuously kept high or rebounded after UAE, while including the only patient who failed on right uterine arterial embolization just mentioned above. Between them five achieved good outcomes, but despite technical success in uterine artery chemembolization together with systematic MTX administration, persistently growing gestational sacs were found in four patients three weeks later by transvaginal ultrasound. Finally hysterectomy was inevitably performed to remove the fetus. 

Thus the rate of total uterine preservation was 82.6% (19/23).

## 4. Discussion

Originally uterine artery embolization is an effective treatment for hysteromyoma [9]. For its high technical successful rate, few complications, and capability to preserve fertility, it is also widely used in symptomatic hysteromyoma, arteriovenous malformation, colporrhagia, and various intractable complications following gynecologic operation (certainly including caesarean section). The writer has previously reported its application to one case of intravenous leiomyoma [10].

MTX has direct cytotoxic effect on chorionic villous tissue. After intra-arterial MTX infusion and subsequent transcatheter arteries embolization, blood flow is stemmed in the uteroplacental arteries. As a result adherent placental tissue and trophoblasts in the sectional scar cannot survive due to both drug killing effect and regional ischemia [11]. That is the reason of uterine artery chemembolization application together.

In this case series, all the interventional operations except one were technologically successful, but finally four patients underwent abdominal hysterectomy and lost their uteri. This result is consistent with other studies [4], suggesting that uterine artery MTX infusion and embolization were not always effective.

Several factors are likely to lead to failure to preserve the patients' fertility and need to pay attention in the clinic work.

The administration of MTX is the first important factor. Because this kind of pregnancy is surrounded by fibrous scar rather than normally vascularized myometrium, slow drug absorption into the CSP is predictable after the administration of systemic MTX [12]. 

At the early stage of our study first eight patients received a single dose (1 mg/kg) of intramuscular MTX in the ward before UAE. During the interventional operation we reduced MTX dosage to 50 mg after prudently attempt to avoid nephrotoxicity and other toxic side-effect of excessive MTX. Among them, two patients had continuous growth of the embryos despite together with following MTX intramuscular administration and finally underwent hysterectomy. 

From feedback in these early cases, we thought that since uterine arterial DSA imaging showed the gestational sac clearly, target artery MTX bolus injection should significantly increase the peak concentration within the fetus in the scar, prolong the local action time, and increase drug bioavailability. Considering that we had been sufficiently communicated with the gynecologists in our hospital. 

The following patients no longer received MTX administration before the DSA operations. The total dose of MTX infusion reached 100–120 mg in some cases. If serum *β*-hCG levels were still high or rebounded after interventional radiology procedure, subsequently local MTX injection was given. Now this management protocol is the current plan of CSP treatment in our hospital. 

Alternative drug, dactinomycin (0.5 mg), is recommended to infuse into uterine artery of the literature [13].

Selection of embolic material is also a significant factor. Gelatin sponge is currently regarded as temporary embolization agent (2–6 week duration of occlusion). In the early period of this study gelatin sponge was cut by hand which often produced various sizes of the particles until standard size (350 *μ*m–560 *μ*m) foam particles (Alicon, Hangzhou, China) had been available since 2011. As an UAE routine procedure, postembolization angiography was applied to confirm the completeness of occlusion of the blood supply to the targeted region. Gelatin sponge particles especially cut by hand on the operating table were actually insufficient for producing a satisfactory vessel-occluding effect and adequate devascularization. Especially if injected at a high speed, gelatin sponge is much more easily to block the arterial trunk than arterioles.

It is probably more effective to use polyvinyl alcohol (PVA), embosphere microspheres, and other permanent materials in comparison with absorbable sponge particles [11]. However, great concern has been raised for the risk that PVA or embosphere microspheres may induce nontarget embolization via abundant veins which results in uterine necrosis and other severe complications [14, 15].

Ultimately, exact identification of target artery is another critical success factor in ensuring the efficacy of transcatheter arterial chemoembolization. In most cases, it can be clearly determined through bilateral uterine artery angiograms which show contour of gestational sac in the pelvis. However, due to recanalization and collateral circulation of the embryo, the blood flow may not be completely stopped by embolization of the uterine arteries only [16]. An experienced interventional radiologist may notice some indistinct signs such as deficiency of blood supply distribution. Ovarian arteries stemming from abdominal aorta sometimes even superior epigastric arteries also supply the gestational sac in the scar that might be neglected by a rough analysis of the angiography.

## 5. Conclusions

To deal with the rising trend of incidence of cesarean scar pregnancy, it should be emphasized to pay particular attention to avoid unnecessary abdominal deliveries in low-risk primiparae [17]. Once the diagnosis of CSP is confirmed, the best choice is to terminate the pregnancy as soon as possible. The sooner, the better.

It is true that until now, no universal guidelines have been established, and individual therapy is strongly recommended. The management protocol may vary according to the symptoms, gestational age, variable fetus, and peritrophoblastic vascularization. 

To sum up, our study suggested that uterine artery embolization combined with target artery MTX infusion is effective to treat high-risk CSP especially complicated with severe colporrhagia in preference to hysterectomy. After all, immediate hemostasis and potentiated cytocidal effects achieved by UAE and chemotherapeutic drug infusion of target area secure sufficient time for the patient and medical team to consider the subsequent treatment strategy [13]. 

Three factors are central to a successful outcome in the interventional treatment: adequate MTX dosage, appropriate embolic material, and complete embolization of target arteries which supply blood to embryo. In some complicated cases, subsequent systemic MTX injection, D&C or laparotomy and excision are regarded as important measures after DSA operation. Hysterectomy may be required in cases of placental abnormalities and undetectable bleeding foci [18].

Individualized treatment respecting actual status of each patient should be the key principle of therapeutic approach toward CSP, and combination therapy probably has higher successful rate of preserving the future fertility.

## 6. Synopsis

Transcatheter arterial chemoembolization is effective to treat cesarean scar pregnancy, but three factors should be highlighted: adequate methotrexate dosage, appropriate material, and complete embolization.

## Abbreviations

CSP: Cesarean scar pregnancy
MTX: Methotrexate
UAE: Uterine artery embolization
D&C: Dilatation and curettage
*β*-hCG: *β*-human chorionic gonadotropin
MRI: Magnetic resonance imaging.
